# 4137 Discrimination, Social Cohesion, and Hypertension: A Cross-Sectional Analysis from the REGARDS Study

**DOI:** 10.1017/cts.2020.384

**Published:** 2020-07-29

**Authors:** Zachary H McCann, April Carson, Verna Keith, Timothy Plante, Rachel Stuckwisch, Suzanne Judd

**Affiliations:** 1 University of Alabama at Birmingham

## Abstract

OBJECTIVES/GOALS: This research has two goals. The first is to establish the relationship between hypertension, discrimination, and social cohesion in the general populaiton. The hypotheses for these relationships are:

- **Hypothesis 1**: Self-reported discrimination will be positively associated with incidence of hypertension in all REGARDS participants.
- **Hypothesis 2**: Self-reported neighborhood cohesion will attenuate the effects of discrimination on the development of hypertension.
- **Hypothesis 3**: Increased levels of Self-reported social cohesion will moderate the effects of discrimination on the likelihood of being hypertensive by weakening the relationship between discrimination and the likelihood of being hypertensive.

The second goal of this research is examining the the nexus of race and social cohesion, to understand if racial effects exist. The hypotheses for this goal are:

- **Hypothesis 4:** Self-reported discrimination will be positively associated with prevalent hypertension in black REGARDS participants over and above the association between discrimination and hypertension in white REGARDS participants.
- **Hypothesis 5:** Increased levels of self-reported social cohesion for will not moderate the effects of discrimination on the likelihood of being hypertensive by weakening the relationship between discrimination and the likelihood of being hypertensive as strongly for black REGARDS participants as it does for white REGARDS participants

METHODS/STUDY POPULATION: The population under investigation will be respondents to The Reasons for Geographic and Racial Differences in Stroke (REGARDS) study at UAB. REGARDS participants are 45+ years old, and come from across the United States. The study will use the second wave of in-home data to assess relationships between discrimination, hypertension, and social cohesion. First we will calculate descriptive statistics (means and standard deviations or N and %) for each of the main variables and covariates. Modified Poisson regression will be used to model the association between discrimination and likelihood of hypertension, as well as the moderating effects of neighborhood social cohesion. We will run additional modified Poisson models including 1) demographic covariates (age, gender, race, rurality, education), 2) demographic covariates plus lifestyle factors, such as smoking status, BMI, exercise frequency, and 3) demographic covariates, lifestyle factors, and social characteristics, including insurance status, social support, getting around. To understand the main effect of social cohesion on hypertension, we will run a second set of models, following the same series of steps. A final series of models will test the interaction between perceived discrimination*perceived neighborhood cohesion. This model will be followed by the same series of steps as the global hypertension models. If the interaction is significant, postestimation will be used to model how different levels of perceived discrimination and cohesion are expected to interact to affect the likelihood of hypertension. If any of the covariates violates assumptions about distributions, data transformations will be explored. After conducting an analysis on the effects of discrimination on hypertension, and the buffering effect of social cohesion on the full sample population, the sample will be stratified by race so that the associations between discrimination, social cohesion, hypertension, and race are fully explored, consistent with previous literature. The stratified samples will be run through the same series of models as the full sample, providing information on how race, hypertension, and social cohesion are associated in and unadjusted models, models adjusted for demographic characteristics, models adjusted for sociodemographic and lifestyle characteristics, and finally full models that adjust for sociodemographic, lifestyle, and social characteristics. These models will compare each of these sets of characteristics, as well as the interaction between perceived discrimination* perceived neighborhood cohesion for stratified samples. This will allow researchers to compare the effects of discrimination, and the buffering effect of neighborhood cohesion across models. RESULTS/ANTICIPATED RESULTS: We anticipate that in a general population, social cohesion will attenuate the positive association of discrimination and hypertension. For blacks, however, higher levels of lifetime discrimination as well as lower levels of social cohesion in primarily black neighborhoods are expected to lead to both higher rats of hypertension and a lower degree of attenuation of the relationship between discrimination and hypertension. DISCUSSION/SIGNIFICANCE OF IMPACT: This study will help to elucidate the social nature of hypertension. By examining how perceptions of discrimination and social cohesion are associated with hypertension, interventions directed at improving heart health will be able to be effectively implemented at the community level by targeting specific social factors that can improve health outcomes. In addition, this research will provide insight into groups that may be particularly vulnerable to hypertension, advancing both the sociological and cardiology literature as it relates to discrimination, social cohesion, race and health.

